# Association between egg consumption and elevated fasting glucose prevalence in relation to dietary patterns in selected group of Polish adults

**DOI:** 10.1186/s12937-019-0516-5

**Published:** 2019-12-30

**Authors:** Anna Czekajło-Kozłowska, Dorota Różańska, Katarzyna Zatońska, Andrzej Szuba, Bożena Regulska-Ilow

**Affiliations:** 1 Department of Dietetics, Wroclaw Medical University, Parkowa 34, 51-616 Wrocław, Poland; 2 Department of Social Medicine, Wroclaw Medical University, Wrocław, Poland; 3 Department of Angiology, Hypertension and Diabetology, Wroclaw Medical University, Wrocław, Poland

**Keywords:** Egg intake, Fasting glucose, Dietary patterns, PURE study, Food frequency questionnaire

## Abstract

**Background:**

The safety of high egg intake in view of its impact on glucose metabolism remains inconclusive. The aim of the study was to assess the relationship between egg intake, dietary patterns and elevated fasting glucose (FG) level in a selected group of Polish adults.

**Methods:**

The study group consisted of 1630 adults who participated in the Polish arm of the Prospective Urban Rural Epidemiological Study. Dietary intake, including egg intake, was assessed based on the data from the Additional file 2 Food Frequency Questionnaire previously validated for the population of Lower Silesia. DPs were derived using principal component analysis. FG levels ≥100 mg/dl were considered elevated. Subjects who used antidiabetic drugs were included in the group with elevated FG levels.

**Results:**

Egg consumption increased in higher quartiles of “Western” and “traditional” DPs in both men and women (*p* < 0.0001). In a crude model each 10 g of eggs consumed per day was associated with 7% increased risk (OR 1.07; 95% CI: 1.01–1.15) of elevated glucose level in the overall group and 10% increased risk (OR 1.10; 95% CI: 1.01–1.21) of elevated glucose level in the group of men. Men who consumed at least five eggs per week had higher risk for elevated FG level compared to men who consumed at most one egg per week (OR 1.79; 95% CI 1.13–2.84), but this relationship became insignificant when the data were adjusted for DPs. In the group of women no association between egg intake and elevated FG level was observed.

**Conclusions:**

Higher egg intake may be associated with the overall unhealthy dietary habits, which is why the number of eggs consumed daily should not be considered an independent risk factor for elevated fasting glucose level.

## Introduction

Eggs are important source of protein and nutrients in the daily diet. An egg weighing 50 g fulfills 12% of daily protein requirement and contains relatively high amounts of monounsaturated fatty acids, iron, zinc, vitamin A and vitamin B_12_. However, egg yolks are also a source of cholesterol and saturated fatty acids (SFA). Two eggs contain 360 mg of cholesterol and 3 mg of SFA which provide about 1,4% of average daily energy intake [1]. The content of the last two components in egg yolk is actually the reason for numerous controversies about frequent egg consumption. In fact, despite many studies conducted on various populations, the association between eggs or dietary cholesterol intake and cardiovascular risk factors remains inconclusive. It seems that SFA, rather than dietary cholesterol, are responsible for elevated blood lipid level [2]. Nevertheless, the effect of cholesterol consumption may depend on individual predispositions and may be related to current health status and genetic factors [3].

Dietary guidelines concerning the recommended cholesterol intake has been changed over the years. In 2006 American Heart Association recommended to consume at most 300 mg of dietary cholesterol per day [4]. However, in “2007 European Guidelines on cardiovascular disease prevention” and in “2015 – 2020 Dietary Guidelines for Americans” no recommendation for dietary cholesterol intake was mentioned [5, 6]. Current Polish recommendations also do not set up the upper limits for cholesterol intake but emphasize the need to substitute food products rich in SFA and cholesterol by those containing unsaturated fatty acids. According to the Polish authors, healthy individuals with normal cholesterol level in blood may thus consume up to seven eggs per week. However, for subjects with T2DM (type 2 diabetes mellitus) the recommendations are more restrictive, and it is suggested to limit egg intake up to two per week [7]. Overall dietary cholesterol restriction is advised for diabetic patients [8].

The safety of high egg intake in view of its impact on glucose metabolism was analyzed in many studies. Djoussé et al. [9] in a prospective study of 20,703 men from the Physicians’ Health Study and 36,295 women from the Women’s Health Study observed that daily egg consumption was associated with an increased risk of T2DM (7 eggs per week vs. no egg consumption: OR 1.77; 95% CI 1.28–2.43 in women and OR 1.58; 95% CI 1.25–2.01 in men). In a meta-analysis performed by Li et al. [10], the relative risk (RR) of diabetes was 1.68 (95% CI 1.41–2.0) for the highest vs. the lowest egg intake and 1.29 (95% CI 1.21–1.37) for each 4 per week increment in egg intake. Higher egg consumption was associated with the increased risk of coronary heart disease and mortality in a group of adult diabetics [11–13]. In a meta-analysis of prospective studies based on the US data Djoussé et al. [14] observed no relationship between infrequent egg consumption and T2DM risk, however there was a modest elevated risk of T2DM among individuals consuming ≥3 eggs/week. The authors explained that the negative effect of egg consumption may be caused by trimethylamine-N-oxide (TMAO) but there was no enough evidence to confirm that theory. Eventually it was suggested that not only egg consumption, but also dietary patterns should be taken into account in future analysis.

Dietary pattern analysis is used in order to assess the cumulative effect of the single components of the diet on health [15, 16]. Due to various interactions between individual components of the diet, different effect of intake of, for instance, seven eggs per week should be observed in individuals having healthy dietary habits (i.e. consuming lots of vegetables, fruits and whole grains) and those having overall unhealthy diet. Suliga et al. [17] conducted a study to derive the main DPs among Polish adults and to evaluate the association between these patterns and metabolic syndrome and its components. The authors found that higher intake of refined grains, potatoes, sugar and sweets (“Traditional-carbohydrate” DP) was associated with a higher risk of abdominal obesity and elevated triglyceride level, whereas a “Westernized” DP was related to hyperglycemia.

Due to the above-mentioned considerations, the effect of egg intake on glucose metabolism should not be assessed separately but as a part of overall diet. The aim of the study was to assess the relationship between egg consumption and elevated fasting glucose (FG) levels according to the DPs derived in the study group.

## Methods

### Study population

**Fig. 1:**
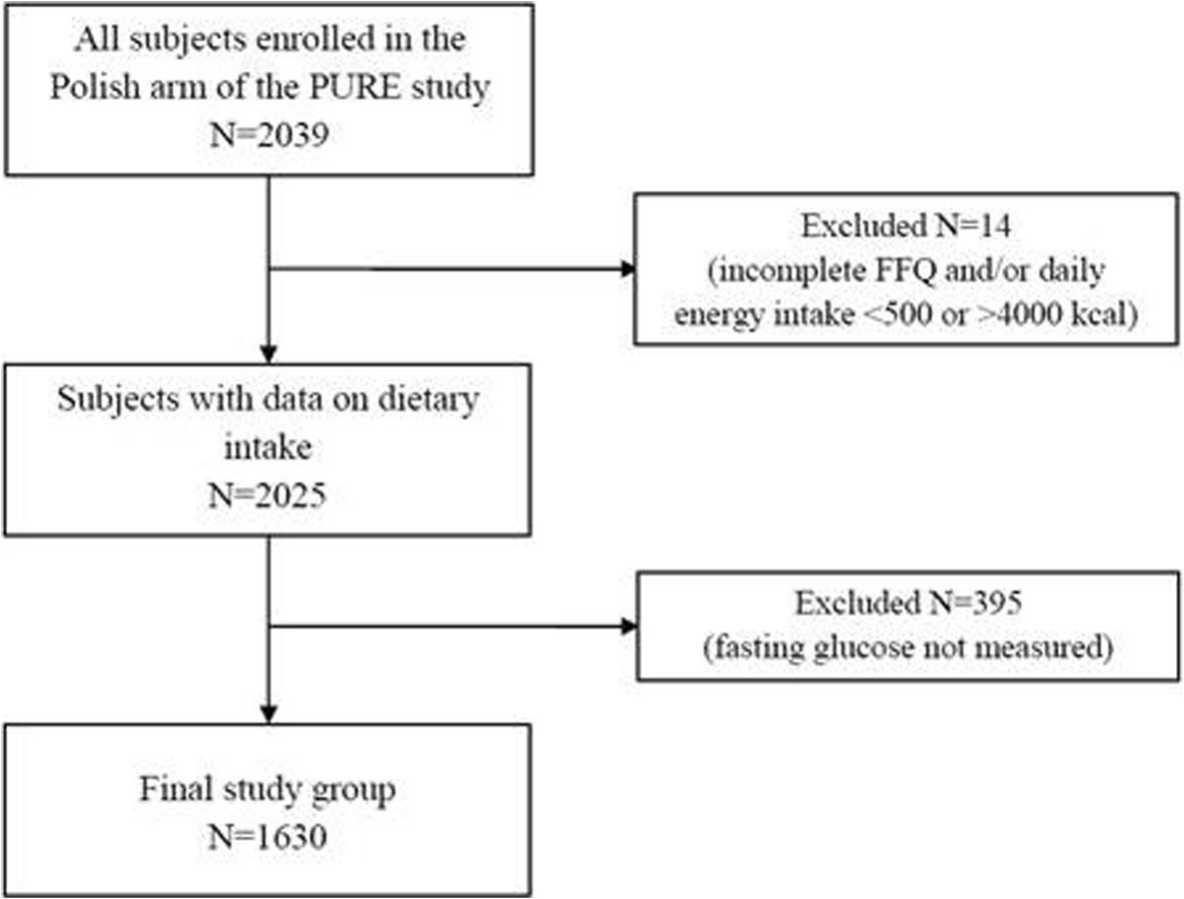
Flow chart of sample collection

Prospective Urban Rural Epidemiological (PURE) Study is an international cohort study which at baseline involved 153,996 adults from 17 countries with different income levels [18]. Individuals were recruited to the Polish arm of the PURE Study through the radio and television announcements. The study was conducted between 2007 and 2009. The general inclusion criteria were as follows: age 35–70 years, permanent residence in the urban or rural areas of the Lower Silesian Voivodeship and daily energy intake between 500 and 4000 kcal. Out of 2025 individuals who were involved in the overall Polish arm of the baseline PURE Study, 1630 adults (1041 women and 589 men) had complete medical data on analyzed factors and were included in the study group. Flow chart of sample collection was presented in Fig. 1. The comparison of the study group and the drop out group was presented in the Additional file 1: Table S1.

### Blood glucose

Blood glucose level was measured in venous blood samples after an overnight fast using the Ascensia Entrust Glucometer (Bayer, Germany). Fasting glucose levels ≥100 mg/dl were considered elevated. Participants who used antidiabetic drugs were automatically included in the group with elevated FG levels.

### Eggs consumption and dietary patterns assessment

As the average consumption of different food products is culture-dependent, dietary intake was evaluated using the data from the Additional file 2 Food Frequency Questionnaire (FFQ) previously validated for the population of Lower Silesia [19]. Frequency of consumption of 154 food products and dishes in 12 months preceding the study was recorded in nine different categories: “never or less than once a month”, “1-3 times a month”, “once a week”, “2-4 times a week”, “5-6 times a week”, “once a day”, “2-3 times a day”, “4-5 times a day” or “more than 6 times a day”. “Album of photographs of food products and dishes” was used in order to estimate the average portion sizes of consumed foods [20]. Dietary patterns were identified in the study group a posteriori using a principal component analysis with varimax rotation. This statistical method aims to identify the main patterns of food consumption, explaining as much of the data variance as possible. Food products from the FFQ were categorized into 22 groups based on their nutritional value and usual consumption purpose. Products that are not a source of energy, such as unsweetened coffee and tea, were not included in the analysis. Characteristics of the food groups were summarized in Table 1.

**Table 1:** Characteristics of the food groups used in the dietary pattern analysis

Food group	Products
Milk and low-fat dairy	Low-fat milk, 1–2% fat [1], milk, 3.2% fat [2], buttermilk, 0.5% fat [3], cocoa with milk [4], cottage cheese [6], quark, fresh cheese [10], low-fat yoghurt [11], yogurt, 2–8% fat [12], kefir [13]
High-fat cheese and cream	Feta cheese [5], hard cheese [7], cheese, “fromage” naturel [8], cheese, Edam type, fat [9], cream, 12% fat [14], cream, 18% fat [15]
Fats	Margarine, soft [16], butter [18], lard [19], Finea/Masmix [20], mayonnaise [148]
Fruits	Apple [21], banana [22], grapefruit [23], grapes [24], tangerine [25], strawberries [26], kiwi fruit [27], lemon [28], orange [29], peach [30], pear [31], plum [32], raspberries [33]
Vegetables	Beans, white (boiled) [34], beets, cooked [35], broccoli, green [36], cabbage, red (raw) [37], cabbage, Shantung [38], cabbage, white (raw) [39], cabbage, white (boiled) [40], carrot (fresh) [41], carrot (boiled) [42], cauliflower (raw) [43], cauliflower (boiled with butter) [44], chives [45], cucumber (raw) [46], garlic (raw) [47], lettuce [48], mushroom (fried) [49], onion (raw) [50], parsley, leaves [51], horseradish [52], pepper (cooked) [53], pepper, red (raw) [54], radish [58], tomato (raw) [59], tomato (cooked) [60], tomato sauce [61], spinach (cooked) [62], squash, summer (cooked) [63], string beans (boiled) [64], sweet corn (canned, drained) [65], peas green (canned, drained) [66], shantung cabbage, salad with mayonnaise [115], sauerkraut salad [116], lettuce with sour cream salad [118]
Chips	Potato (French fried) [55]
Potatoes	Potato (boiled) [56], potato (mashed) [57]
Red meat	Beef steaks [68], pork, belly (no bone, boiled) [79], pork cutlets (breaded, fried) [81], organ meat (liver, tongue, heart) [89], beef and pork minced cutlets (fried) [100]
Processed red/mixed meat	Beef, ham (cooked) [69], Frankfurter/Hotdog [76], luncheon meat (pork) [77], pork ham [80] sausage Slaska (pork, cooked) [82], sausage Krakowska (pork and beef) [83], sausage Biala (pork) [84], sausage Szynkowa (turkey) [87], Head Cheese, white and black [88] chicken pate (canned) [104]
Low-fat poultry	Chicken without skin (cooked/fried) [73], turkey (roasted) [86]
High-fat/processed poultry	Chicken fillets (breaded, fried) [70], chicken ham [71], chicken with skin (cooked/fried) [72], turkey, ham [85]
Fish	Cod fillets (breaded and fried) [74], herring in cream [75], mackerel (smoked) [78]
Unrefined grains	Rye, brown bread [92], wheat-rye bread with sunflower seeds [96], pasta (cooked) [98], buckwheat groats (boiled) [102], pearl barley groats (boiled) [105], soup, milk with rolled oats [109]
Refined grains	Wheat bread [90], rice (boiled) [91], wheat rolls (kajzerki) [93], wheat rolls (wroclawskie) [94], wheat-rye bread/white bread [95], cold cereal (cornflakes) [97]
Mixed dishes	Baked beans with meat [99], cabbage leaves, stuffed [103], Polish dumplings, with meat [113], sauerkraut with sausage and meat (bigos, stewed) [114], dumplings with potato filling (Ruskie, boiled) [117], vegetable salad (cooked with mayonnaise) [119]
Soups	Broth [101], soup with vegetables [106], soup, Krupnik with pearl barley groats [107], soup, Zurek sour rye [108], soup, tomato [110], soup, sauerkraut [111], soup, white bean [112]
Juices	Orange juice [120], carrot juice [122], apple juice [123], grapefruit juice [124], blackcurrant juice [125], multifruit juice from Polish fruits [126], multifruit juice from exotic fruits [127]
Beverages	Raspberry juice [121], fruits drink [128], soft drink (regular) [129], soft drink (low calorie) [130]
Alcohol	Beer [134], red wine [135], vodka [136]
Sweets	Ice cream [17], milk chocolate [137], bitter chocolate [138], biscuits [139], yeast cake [140], short-cake [141], gingerbread cake [142], sponge cake [143], cheesecake (Krakowski) [144], halva with vanilla [146], drops [147], sweets [151]
Sugar and honey	Honey [145], sugar [152]
Nuts, seeds and raisins	Nuts [149], raisins, dried [150], seeds [153], walnuts [154]

Number in bracket refers to the item number in the Food Frequency Questionnaire (available in Additional file 2)

Due to the fact that in the preliminary analysis eggs did not have high factor loadings for any of identified DPs (so their consumption was not particularly characteristic for any of the dietary patterns), they were not included in the final analysis. The number of patterns was determined based on the eigen values (Kaiser’s criterion), the scree plot and the interpretability of derived factors. As a cut-off point were accepted factor loadings higher than 0.50.

### Statistical analysis

All statistical analyses were conducted using the Statistica software version 12.0 PL (Statsoft Inc., USA). Mean nutritional value was estimated in the diets of studied men and women. Based on the obtained factor scores, for each of the identified DPs study individuals were divided into quartiles or included into one of two following groups: below the median (<Me) or equal/above the median (≥Me) for the gender. The differences in usual egg intake between the quartiles of dietary patterns and categories of egg intake were assessed using the ANOVA Kruskal-Wallis test. Chi-square test for trend was used to evaluate the relationship between usual egg intake and the prevalence of elevated FG level with reference to identified DPs. In order to assess the ORs for the elevated FG level occurrence according to egg intake, logistic regression was applied. Consumption of no more than one egg per week was considered the reference category of egg intake. Six models were created for the overall group and separately for women and men: first model was based on the crude data, while other models were adjusted for age, body mass index (BMI), percentage of energy from SFA, the intake of simple sugars per 1000 kcal and factor scores for identified dietary patterns. The level of statistical significance for all analyses was set at α = 0.05.

## Results

The general characteristics of the study group were presented in Table 2. Mean age and percentage of individuals with elevated blood glucose level was similar in the groups of women and men (54.7 ± 9.8 years vs. 54.1 ± 10.0 years and 37.6% vs. 41.6%, respectively). Men consumed more eggs per day in comparison with women (18.1 ± 18.5 g vs. 14.8 ± 12.6 g; *p* = 0.0084). The percentage of individuals who consumed ≤1 egg/week was similar in both groups. Significantly more women than men consumed 2–4 eggs/week (*p* = 0.0077), but significantly more men than women consumed ≥5 eggs/week (*p* < 0.0001). No difference in energy intake and percentage of energy from SFA was found between gender groups. The intake of cholesterol was higher in the group of men compared to women (302.5 ± 147.7 mg vs. 280.6 ± 130.2 mg; *p* = 0.0130), however after adjustment for energy intake no such relationship was found. Women consumed more simple sugars per 1000 kcal than men (46.7 ± 12.7 vs. 42.1 ± 12.7; *p* < 0.0001).

**Table 2:** General characteristics of the study group (*n* = 1630)

Variable	Women (*n* = 1041)^*^	Men (*n* = 589)^*^	*p*^*#*^
Age [years]	54.7 ± 9.8	54.1 ± 10.0	*NS*
BMI [kg/m^2^]	27.9 ± 5.4	28.8 ± 4.7	*< 0.0001*
% of individuals with blood glucose ≥100 mg/dl	37.6	41.6	*NS*
Egg consumption [g/day]	14.8 ± 12.6	18.1 ± 18.5	*0.0084*
% of individuals who consume ≤1 egg/week	45.9	43.8	*NS*
% of individuals who consume 2–4 eggs/week	45.3	38.5	*0.0077*
% of individuals who consume ≥5 eggs/week	8.7	17.7	*< 0.0001*
Energy intake [kcal/day]	2079.0 ± 757.1	2130.9 ± 718.3	*NS*
SFA intake [% of energy]	12.5 ± 3.4	12.3 ± 4.5	*NS*
Cholesterol intake [mg/day]	280.6 ± 130.2	302.5 ± 147.7	*0.0130*
Cholesterol intake [mg/1000 kcal/day]	134.6 ± 37.7	139.9 ± 42.7	*NS*
Simple sugars intake [g/1000 kcal/day]	46.7 ± 12.7	42.1 ± 12.7	*< 0.0001*

^*^ - values are mean ± standard deviation except for percentage of individuals with elevated blood glucose level and percentage of individuals in particular categories of egg intake; ^**#**^ **-** U-Mann Whitney test for quantitative variables and X^2^ test for qualitative variables; BMI – body mass index; SFA – saturated fatty acids; NS – no statistically significant difference

Three main DPs were identified in the study group. “Western” DP was associated with high factor loadings for fats (0.65), sweets (0.62), refined grains (0.60), high-fat cheese and cream (0.60), processed red/mixed meat (0.59) and sugar & honey (0.55), “prudent” pattern was related to high consumption of fruits (0.71), vegetables (0.66), nuts, seeds & raisins (0.61) and milk and low-fat dairy (0.52), while “traditional” pattern was linked with high intake of mixed dishes (0.75), soups (0.69), red meat (0.61) and fish (0.59). Together derived DPs explained 36.7% of the total variance. The factor-loading matrix for the identified DPs was presented in Table 3.

**Table 3:** Factor-loading matrix for dietary patterns identified in the study group^*^

Variable	“Western” DP	“Prudent” DP	“Traditional” DP
Fats	0.65		
Sweets	0.62		
Refined grains	0.60		
High-fat cheese and cream	0.60		
Processed red/mixed meat	0.59		
Sugar & honey	0.55		
Chips			
Juices			
Fruits		0.71	
Vegetables		0.66	
Nuts, seeds and raisins		0.61	
Milk and low-fat dairy		0.52	
Unrefined grains			
Beverages			
Mixed dishes			0.75
Soups			0.69
Red meat			0.61
Fish			0.59
Low-fat poultry			
High-fat/processed poultry			
Potatoes			
Alcohol			
Percentage of variance explained (%)	19.82	10.57	6.27

^*^ the absolute values of factor loadings ≥0.5 were shown; DP - dietary pattern

Both in the group of women and men, egg intake increased in higher quartiles of “Western” and “traditional” DPs (*p* < 0.0001). No association was observed with reference to “prudent” pattern. The similar association was observed in the overall study group (Table 4).

**Table 4:** Egg intake (g per day) in quartiles of derived dietary patterns (X ± SD) in group of women and men

Group	Q of DP	“Western” DP		“Prudent” DP		“Traditional” DP	
		Egg intake (g/day) X ± SD	p^*^	Egg intake (g/day) X ± SD	p^*^	Egg intake (g/day) X ± SD	p^*^
Women (n = 1041)	Q1	11.9 ± 11.9	*< 0.0001*	13.3 ± 9.8	*NS*	10.8 ± 9.2	*< 0.0001*
	Q2	13.4 ± 12.0		15.6 ± 13.2		14.3 ± 11.5	
	Q3	14.8 ± 12.8		14.8 ± 13.4		16.7 ± 11.7	
	Q4	19.0 ± 12.8		14.8 ± 13.6		17.4 ± 16.0	
Men (n = 589)	Q1	11.4 ± 14.7	*< 0.0001*	20.4 ± 21.8	*NS*	12.9 ± 12.9	*< 0.0001*
	Q2	14.7 ± 13.2		17.8 ± 17.9		16.3 ± 15.3	
	Q3	18.5 ± 16.4		18.1 ± 19.2		20.5 ± 18.6	
	Q4	27.5 ± 23.7		16.0 ± 13.9		22.5 ± 23.8	
Overall (n = 1630)	Q1	11.7 ± 12.1	*< 0.0001*	16.6 ± 16.6	*NS*	10.9 ± 9.5	*< 0.0001*
	Q2	14.0 ± 13.3		15.8 ± 15.5		15.7 ± 13.6	
	Q3	16.0 ± 13.5		15.8 ± 12.5		17.6 ± 14.6	
	Q4	22.2 ± 18.5		15.8 ± 15.3		19.7 ± 19.5	

^*^ - ANOVA Kruskal-Wallis test; X ± SD – mean ± standard deviation; DP – dietary pattern; Q1, Q2, Q3, Q4 – quartile 1, 2, 3, 4; NS – no statistically significant difference

Table 5 shows the comparison of dietary cholesterol intake and percentage of energy from SFA depending on habitual egg intake in the study group. Increased egg intake was associated with higher cholesterol intake per 1000 kcal and higher percentage of energy from SFA in the overall study group and in both men and women (*p* < 0.0001). When the intake of SFA and cholesterol from eggs were not taken into account in the analysis the results remained similar. The intake of cholesterol increased from 99.5 ± 25.6 mg/1000 kcal in the group who consumed ≤1 egg per week to 123.6 ± 27.4 mg/1000 kcal in the group who consumed ≥5 eggs per week (*p* < 0.0001). The percentage of energy from SFA increased from 11.6 ± 3.4% to 13.5 ± 3.4% (*p* < 0.0001), respectively.

**Table 5:** Comparison of dietary cholesterol intake and percentage of energy from saturated fatty acids (X ± SD) depending on habitual egg intake in the study group

Dietary intake	Women (*n* = 1041)				Men (n = 589)				Overall (n = 1630)			
	≤ 1 egg per week	2–4 eggs per week	≥ 5 eggs per week	p^*^	≤ 1 egg per week	2–4 eggs per week	≥ 5 eggs per week	p^*^	≤ 1 egg per week	2–4 eggs per week	≥ 5 eggs per week	p^*^
Cholesterol [mg/1000 kcal/day]	109.8 ± 27.1	149.3 ± 25.6	188.1 ± 42.9	*< 0.0001*	110.5 ± 26.7	147.8 ± 26.9	195.7 ± 39.9	*< 0.0001*	110.1 ± 26.9	148.8 ± 26.0	192.1 ± 41.4	*< 0.0001*
Cholesterol other than from eggs [mg/1000 kcal/day]	99.3 ± 25.7	114.5 ± 25.6	119.5 ± 28.9	*< 0.0001*	100.1 ± 25.7	112.9 ± 27.6	127.3 ± 25.6	*< 0.0001*	99.5 ± 25.6	114.0 ± 26.3	123.6 ± 27.4	*< 0.0001*
SFA [% of energy]	11.9 ± 3.5	12.9 ± 3.2	13.9 ± 3.5	*< 0.0001*	11.5 ± 3.4	12.5 ± 3.4	14.1 ± 3.4	*< 0.0001*	11.7 ± 3.4	12.8 ± 3.3	14.0 ± 3.4	*< 0.0001*
SFA other than from eggs [% of energy]	11.8 ± 3.5	12.7 ± 3.3	13.3 ± 3.5	*< 0.0001*	11.4 ± 3.4	12.3 ± 3.4	13.6 ± 3.4	*< 0.0001*	11.6 ± 3.4	12.5 ± 3.3	13.5 ± 3.4	*< 0.0001*

^*^ **-** Kruskal-Wallis test; SFA – saturated fatty acids X ± SD - mean ± standard deviation

In the group of women egg intake was not associated with the prevalence of elevated FG level, regardless of dietary pattern. Instead, higher prevalence of elevated fasting glucose level was observed in the group of men who had high factor scores for “Western” or “traditional” DPs (≥Me for the group; *p* for trend 0.0403 and 0.0045, respectively). Egg consumption was not associated with elevated FG in the group of men who had overall “prudent” DP (≥Me for the group; *p* for trend 0.2060) (data not shown in tables).

The ORs and 95% confidence intervals of the elevated glucose level occurrence in relation to egg consumption in the study group were presented in Table 6. In a model based on the crude data, men who consumed at least five eggs per week had higher odds ratio for elevated FG level compared to men who consumed at most one egg per week (OR 1.79; 95% CI 1.13–2.84). The relationship remained statistically significant after adjustment for age and BMI (model 2: OR 1.71; 95% CI 1.05–2.77) and age, BMI, percentage of energy from SFA and the intake of simple sugars per 1000 kcal (model 3: OR 1.66; 95% CI 1.01–2.74). However, when the data were adjusted also for the factor scores for derived dietary patterns (“Western” DP – model 4, “prudent” DP – model 5 and “traditional” DP – model 6), no relationship between the category of egg consumption and elevated FG level occurrence was observed. In the group of women, egg intake was not linked with the elevated FG level in any of the studied models. Taking into account the overall study group, in a model based on the crude data, the consumption of at least five eggs per week was associated with higher odds ratio for elevated FG level compared to the consumption of at most one egg per week (OR 1.39; 95% CI 1.03–1.89), however the relationship did not remain statistically significant after adjustment for confounding variables. For each increase in egg intake of 10 g/day (crude data) in the group of men there was a 10% increased risk (OR 1.10; 95% CI 1.01–1.21) of elevated glucose level, while in the overall study group there was a 7% increased risk (OR 1.07; 95% CI 1.01–1.15) of elevated glucose level. After adjustment for confounding variables the relationships did not remain statistically significant.

**Table 6:** The odds ratio (OR) and 95% confidence interval (95% CI) of the elevated glucose level (≥ 100 mg/dl) in relation to habitual egg consumption in the study group

Group	Category of habitual egg intake	OR (95% CI)					
		Model 1	Model 2	Model 3	Model 4 (*Western DP*)	Model 5 (*Prudent DP*)	Model 6 (*Traditional DP*)
Women (*n* = 1041)	≤1 egg/week	1.00	1.00	1.00	1.00	1.00	1.00
	2–4 eggs/week	0.92 (0.71–1.21)	0.87 (0.66–1.14)	0.87 (0.66–1.15)	0.81 (0.61–1.07)	0.87 (0.66–1.15)	0.86 (0.65–1.13)
	≥5 eggs/week	1.06 (0.67–1.67)	1.06 (0.66–1.71)	1.06 (0.65–1.71)	0.99 (0.62–1.59)	1.09 (0.67–1.78)	1.01 (0.65–1.56)
	10 g increased egg intake/day	1.03 (0.93–1.14)	1.02 (0.92–1.13)	1.02 (0.92–1.13)	1.00 (0.90–1.10)	1.02 (0.92–1.14)	1.01 (0.91–1.12)
Men (*n* = 589)	≤1 egg/week	1.00	1.00	1.00	1.00	1.00	1.00
	2–4 eggs/week	1.17 (0.81–1.69)	1.07 (0.73–1.57	1.06 (0.72–1.56)	0.97 (0.65–1.44)	1.07 (0.72–1.57)	1.05 (0.71–1.55)
	≥5 eggs/week	**1.79 (1.13–2.84)**	**1.71 (1.05–2.77)**	**1.66 (1.01–2.74)**	1.32 (0.77–2.28)	1.65 (0.99–2.72)	1.61 (0.97–2.67)
	10 g increased egg intake/day	**1.10 (1.01–1.21)**	1.08 (0.98–1.19)	1.07 (0.97–1.18)	1.02 (0.92–1.13)	1.07 (0.97–1.18)	1.06 (0.96–1.17)
Overall^*^ (n = 1630)	≤1 egg/week	1.00	1.00	1.00	1.00	1.00	1.00
	2–4 eggs/week	1.00 (0.97–1.03)	0.94 (0.75–1.17)	0.94 (0.75–1.17)	0.86 (0.68–1.08)	0.94 (0.75–1.17)	0.92 (0.74–1.16)
	≥5 eggs/week	**1.39 (1.03–1.89)**	1.34 (0.96–1.87)	1.33 (0.94–1.87)	1.16 (0.82–1.66)	1.34 (0.95–1.89)	1.28 (0.90–1.81)
	10 g increased egg intake/day	**1.07 (1.01–1.15)**	1.05 (0.98–1.13)	1.05 (0.98–1.12)	1.02 (0.95–1.10)	1.05 (0.98–1.13)	1.04 (0.97–1.12)

DP – dietary pattern; Model 1 – without adjustment for confounding variables; Model 2 – adjusted for age and body mass index (BMI); Model 3 – adjusted for age, BMI, percentage of energy from saturated fatty acids (SFA) and simple sugars intake/1000 kcal; Model 4 - adjusted for age, BMI, percentage of energy from SFA, simple sugars intake/1000 kcal and factor score for “Western” DP; Model 5 - adjusted for age, BMI, percentage of energy from SFA, simple sugars intake/1000 kcal and factor score for “prudent” DP; Model 6 - adjusted for age, BMI, percentage of energy from SFA, simple sugars intake/1000 kcal and factor score for “traditional” DP; ^*^ - all models (exc. Model 1) additionally adjusted for sex

## Discussion

Three main DPs were identified in the study group: “Western”, “prudent” and “traditional”. Egg intake increased in higher quartiles of “Western” and “traditional” DPs, but no association was observed with reference to “prudent” pattern. The association between prevalence of elevated FG and egg intake was observed only in the group of men with higher adherence to “Western” or “traditional” DPs. Men who consumed at least five eggs per week had higher risk for elevated FG level compared to men who consumed at most one egg per week, but in the models adjusted for the factor scores for dietary patterns, no such relationship was observed.

The dietary risk factors for diabetes include refined grains, sugar-sweetened beverages, fruit juices and red and processed meat [21]. Eggs are also considered a dietary component increasing the risk of T2DM especially due to possible negative effect of TMAO which is formed from dietary choline, naturally found in eggs [14]. However, according to the results of the presented study, the assessment of the association between egg consumption and impaired glucose metabolism should not be based solely on the number of eggs consumed weekly. The most common egg dishes contain products with different nutritional characteristics. Eggs may be used to prepare various fried dishes, cakes and dressings which cannot be recommended in the daily diet due to their nutritional value. It also seems incorrect not to mention the difference in the nutritional value of e.g. scrambled eggs made with olive oil and vegetables and scrambled eggs made with butter and bacon, which results from a significantly different fatty acids profile of these two dishes. Moreover, unhealthy dietary habits are usually related to the excessive adiposity which is the major risk factor for diabetes itself [22]. The question is therefore whether eggs themselves may cause harmful health effects or maybe they are usually consumed as a part of unhealthy dietary pattern.

In the herein study, egg intake increased in higher quartiles of “Western” and “traditional” DPs in both men and women. “Western” pattern was characterized by high consumption of food products considered unhealthy. In other studies, this type of dietary pattern was linked with elevated FG levels or diabetes [23–26]. “Traditional” pattern identified in our study had high factor loadings for various food groups. Some of them contain products highly recommended in the daily diet, such as vegetables, grains and oily fish. However, typical Polish dishes are often prepared with the use of butter or lard, important sources of SFA in the diet [1]. Study individuals who consumed more eggs had higher intake of SFA and cholesterol even when the intake of these nutrients from eggs was not included in the analysis. Moreover, it is known that food preparation techniques, including boiling, frying or roasting, may affect the nutritional value of dishes: increase their glycemic index and decrease the content of essential nutrients [27]. Consumption of eggs was not associated with the “prudent” pattern. Obtained results indicate that subjects who consumed more eggs simultaneously had less healthy eating habits so potential metabolic abnormalities could result from their overall dietary habits.

Nevertheless, in the group of women there was no association between the category of egg intake and the elevated FG prevalence neither in the overall group nor in subgroups with the factor scores for the identified dietary patterns below or equal/above the median. Moreover, in the group of women egg consumption was not related to the risk of elevated FG level in any of the created models. Obtained results appear to be consistent with the observations made by Woo et al. [28] who found no association between egg consumption and elevated FG level in a group of adult Korean women. Nonetheless, Shin et al. [29] reported that higher egg consumption was inversely associated with the elevated FG level in women (7 eggs per week vs. < 1 egg per week: OR 0.94; 95% CI 0.83–0.99).

In the group of men, the prevalence of elevated FG level increased across the categories of egg consumption, however the association remained significant only in men in the subgroups ≥ median factor scores for the “Western” and “traditional” DPs. In the group of men who consumed large amounts of eggs but simultaneously had overall healthy dietary habits, egg intake was not linked with elevated FG level. When the logistic regression models were adjusted for the factor scores for the DPs, the increased OR for elevated FG level observed in the crude model in the highest category of egg intake became insignificant, which indicates that in this case the overall eating habits might have played a greater part in the development of this abnormality.

The results of the studies on the relationship between egg intake and elevated FG level among men conducted by other authors are not conclusive. Woo et al. [28] observed that higher egg consumption (> 3 eggs per week) was associated with a decreased risk of elevated FG level (RR 0.39; 95% CI 0.22-0.67). In a study performed by Shin et al. [29] no such relationship was found. Park et al. [30] observed that in a group of individuals who consumed 4–6 eggs per week the OR for elevated FG level was 0.82 (95% CI 0.72–0.93). In this study a gender-stratified statistical analysis was not performed due to a small number of subjects in the categories of egg intake.

Observed differences between results of the studies in both men and women, apart from other lifestyle, socio-economic and genetic factors, may result from different eating habits in various populations. According to the current guidelines, egg may be a part of a healthy DP, as a good source of protein in the diet [6]. However, in some studies eggs were characteristic for unhealthy, usually Western-like dietary patterns, associated with higher risk of hypertriglyceridemia, hypercholesterolemia and obesity [23, 31–34]. In a meta-analysis performed by Jannasch et al. [25] eggs were typical food product for “mainly unhealthy” dietary pattern which was positively associated with T2DM (RR 1.44; 95% CI 1.27–1.62). Although in this study population eggs were not typical for any of the identified patterns in the preliminary analysis, it may be recognized that their consumption together with unhealthy DPs, especially in the group of men, somehow explains obtained results.

Our study has several limitations. Subjects were recruited through radio and television, so the studied group was not a representative sample. Due to the lack of the required data a drop out was higher than 20% what might have affected the results. As it was a cross-sectional study, the causal relationship between egg consumption and elevated FG level could not be assessed and observed relationships should be confirmed in prospective analyses. Another limitation is that we could use only fasting glucose level as a marker of impaired glucose metabolism because it was the only one assessed in the baseline PURE study. However, it is one of the markers most commonly used in epidemiological studies. Nevertheless, the strength of our study is that it was performed in a large group of participants, using standardized methods and country-specific, validated food frequency questionnaire of good quality. Another limitation of our study is the underreporting of habitual food intake especially in men. Although this is a common bias found in nutritional studies, it should be considered when interpreting the results. Patterns identified a posteriori illustrated the actual culture-dependent eating habits of the studied group of subjects. The association between FG level, egg intake and DPs were thus assessed in the light of the interactions between the particular elements of the diet what may provide a new perspective on the studied relationship.

## Conclusions

Higher egg intake may be associated with the overall unhealthy dietary habits, which is why the number of eggs consumed daily should not be considered an independent risk factor for elevated fasting glucose level. The clustering of dietary risk factors may thus explain higher risk of elevated FG observed in the group of men who consume a lot of eggs.

## Supplementary information

**Additional file 1:** **Table S1.** Comparison of the study group and the drop out group.

**Additional file 2:** Adult Semi-Quantitative Food Frequency Questionnaire.

## Data Availability

The datasets used and/or analyzed during the current study are available from the corresponding author on reasonable request.

## Abbreviations

BMI: Body mass index
CI: Confidence interval
CVD: Cardiovascular diseases
DP: Dietary pattern
FFQ: Food frequency questionnaire
FG: Fasting glucose
Me: Median
OR: Odds ratio
PURE Study: Prospective Urban and Rural Epidemiological Study
RR: Relative risk
SFA: Saturated fatty acids
T2DM: Type 2 diabetes mellitus
