# Supplementary material for: Association between egg consumption and elevated fasting glucose prevalence in relation to dietary patterns in selected group of Polish adults
Source: Nutr J. 2019 Dec 30;18:90. doi: 10.1186/s12937-019-0516-5 (PMC6937644; doi:10.1186/s12937-019-0516-5)
Supplement: Supplementary file 1 — Additional file 1: Table S1. Comparison of the study group and the drop out group. [file 12937_2019_516_MOESM1_ESM.docx]

Table S1. Comparison of the study group and the drop out group

| **Variable** | **Study group (n=1630)^*^** | **Drop out group (n=395)^*^** | ***p^#^*** |
| --- | --- | --- | --- |
| Age [years] | 54.5 ± 9.9 | 54.6 ± 9.6 | *NS* |
| Women [%] | 63.9 | 59.7 | *NS* |
| BMI [kg/m^2^] | 28.2 ± 5.2 | 27.8 ± 4.7 | *NS* |
| Eggs consumption [g/day] | 16.0 ± 15.1 | 14.0 ± 15.0 | *0.0002* |
| % of individuals who consume ≤1 egg/week | 45.2 | 57.7 | *<0.0001* |
| % of individuals who consume 2-4 eggs/week | 42.9 | 30.9 | *<0.0001* |
| % of individuals who consume ≥5 eggs/week | 12.0 | 11.4 | *NS* |
| Energy intake [kcal/day] | 2097.8 ± 743.5 | 2062.9 ± 820.4 | *NS* |
| SFA intake [% of energy] | 12.4 ± 3.5 | 12.2 ± 3.5 | *NS* |
| Cholesterol intake [mg/day] | 288.6 ± 137.2 | 264.9 ± 133.1 | *0.0003* |
| Cholesterol intake [mg/1000 kcal/day] | 136.5 ± 39.7 | 129.0 ± 41.4 | *<0.0001* |
| Simple sugars intake [g/1000 kcal/day] | 45.1 ± 12.9 | 45.1 ± 13.5 | *NS* |

^*^ - values are mean ± SD except for percentage of women and percentage of individuals in particular categories of egg intake; **^#^ -** U-Mann Whitney test for quantitative variables and X^2^ test for qualitative variables; BMI – body mass index; SFA – saturated fatty acids; NS – no statically significant difference
