# Supplementary material for: Association between egg consumption and elevated fasting glucose prevalence in relation to dietary patterns in selected group of Polish adults
Source: Nutr J. 2019 Dec 30;18:90. doi: 10.1186/s12937-019-0516-5 (PMC6937644; doi:10.1186/s12937-019-0516-5)
Supplement: Supplementary file 2 — Additional file 2: Adult Semi-Quantitative Food Frequency Questionnaire. [file 12937_2019_516_MOESM2_ESM.pdf]

We are very grateful to you for your participation in this study. All information given by you will be held in strict confidence, and will be used for the purpose of this study only after removing any personal identifying information.

Adult Semi-Quantitative Food Frequency Questionnaire

INSTRUCTIONS

Please answer EACH question by marking an X in ONE BOX on each line: (unless otherwise instructed)

[X]

OR

By writing number(s) in the spaces provided:

[1] [8]

OR

By specifying the answer on the line(s) provided

October 17, 2007

Subject ID

☐☐	☐☐☐	☐☐☐	☐☐
Centre #	*Community #*	*Household #*	*Subject #*

Subject Initials ☐☐☐

During the past year, on average, how often have you consumed the following foods and drinks? (please check the appropriate box)

**Milk and dairy Products**	**Avg Serving**	**Never, less than once/month**	**1-3/mo**	**1/wk**	**2-4/wk**	**5-6/wk**	**1/day**	**2-3/day**	**4-5/day**	**>6/day**
**1.** Low fat milk (1-2% fat)	1 glass	☐	☐	☐	☐	☐	☐	☐	☐	☐
**2.** Milk (3.2% fat)	1 glass	☐	☐	☐	☐	☐	☐	☐	☐	☐
**3.** Buttermilk, 0.5% fat	1 glass	☐	☐	☐	☐	☐	☐	☐	☐	☐
**4.** Cocoa with milk	1 glass	☐	☐	☐	☐	☐	☐	☐	☐	☐
**5.** Feta cheese	1 slice	☐	☐	☐	☐	☐	☐	☐	☐	☐
**6.** Cottage cheese	1 small plate	☐	☐	☐	☐	☐	☐	☐	☐	☐
**7.** Hard cheese	1 slice	☐	☐	☐	☐	☐	☐	☐	☐	☐
**8.** Cheese “Fromage” naturel	1 slice	☐	☐	☐	☐	☐	☐	☐	☐	☐
**9.** Cheese, Edam type, fat	1 slice	☐	☐	☐	☐	☐	☐	☐	☐	☐
**10.** Quark, fresh cheese	1 slice	☐	☐	☐	☐	☐	☐	☐	☐	☐
**11.** Low fat yogurt	1 Tbs	☐	☐	☐	☐	☐	☐	☐	☐	☐
**12.** Yogurt, 2-8% fat	1 Tbs	☐	☐	☐	☐	☐	☐	☐	☐	☐
**13.** Kefir	1 glass	☐	☐	☐	☐	☐	☐	☐	☐	☐
**14.** Cream, 12% fat	1 Tbs	☐	☐	☐	☐	☐	☐	☐	☐	☐
**15.** Cream, 18% fat	1 Tbs	☐	☐	☐	☐	☐	☐	☐	☐	☐

Subject ID

Centre #	Community #	Household #	Subject #

Subject Initials

During the past year, on average, how often have you consumed the following foods and drinks? (please check the appropriate box)

**Milk and dairy Products Cont'**	**Avg Serving**	**Never, less than once/month**	**1-3/mo**	**1/wk**	**2-4/wk**	**5-6/wk**	**1/day**	**2-3/day**	**4-5/day**	**>6/day**
**16.** Margarine, soft	1 Tsp	☐	☐	☐	☐	☐	☐	☐	☐	☐
**17.** Ice cream	1 bowl	☐	☐	☐	☐	☐	☐	☐	☐	☐
**18.** Butter	1 heaped Tbs	☐	☐	☐	☐	☐	☐	☐	☐	☐
**19.** Lard	1 Tbs	☐	☐	☐	☐	☐	☐	☐	☐	☐
**20.** Finea/Masmix	1 Tsp	☐	☐	☐	☐	☐	☐	☐	☐	☐

During the past year, on average, how often have you consumed the following fruits? (please check the appropriate box)

**Fruits**	**Avg Serving**	**Never, less than once/month**	**1-3/mo**	**1/wk**	**2-4/wk**	**5-6/wk**	**1/day**	**2-3/day**	**4-5/day**	**>6/day**
**21.** Apple	1 medium	☐	☐	☐	☐	☐	☐	☐	☐	☐
**22.** Banana	1 medium	☐	☐	☐	☐	☐	☐	☐	☐	☐
**23.** Grapefruit	1 medium	☐	☐	☐	☐	☐	☐	☐	☐	☐
**24.** Grapes	1 medium bunch	☐	☐	☐	☐	☐	☐	☐	☐	☐
**25.** Tangerine	1 medium	☐	☐	☐	☐	☐	☐	☐	☐	☐
**26.** Strawberries	1/2 cup	☐	☐	☐	☐	☐	☐	☐	☐	☐
**27.** Kiwi fruit	1 medium	☐	☐	☐	☐	☐	☐	☐	☐	☐
**28.** Lemon	1 medium	☐	☐	☐	☐	☐	☐	☐	☐	☐
**29.** Orange	1 medium	☐	☐	☐	☐	☐	☐	☐	☐	☐
**30.** Peach	1 medium	☐	☐	☐	☐	☐	☐	☐	☐	☐
**31.** Pear	1 medium	☐	☐	☐	☐	☐	☐	☐	☐	☐
**32.** Plum	1 medium	☐	☐	☐	☐	☐	☐	☐	☐	☐
**33.** Raspberries	1 glass	☐	☐	☐	☐	☐	☐	☐	☐	☐

Subject ID

☐☐	☐☐☐	☐☐☐	☐☐	**Subject Initials** ☐☐☐
Centre #	*Community #*	*Household #*	*Subject #*	

During the past year, on average, how often have you consumed the following vegetables? (please check the appropriate box)

**Vegetables**	**Avg Serving**	**Never, less than once/month**	**1-3/mo**	**1/wk**	**2-4/wk**	**5-6/wk**	**1/day**	**2-3/day**	**4-5/day**	**>6/day**
**34.** Beans, white (boiled)	1 plate	☐	☐	☐	☐	☐	☐	☐	☐	☐
**35.** Beets, cooked	1 plate	☐	☐	☐	☐	☐	☐	☐	☐	☐
**36.** Broccoli, green	1 plate	☐	☐	☐	☐	☐	☐	☐	☐	☐
**37.** Cabbage, red (raw)	1 plate	☐	☐	☐	☐	☐	☐	☐	☐	☐
**38.** Cabbage, Shantung	1 plate	☐	☐	☐	☐	☐	☐	☐	☐	☐
**39.** Cabbage, white (raw)	1 plate	☐	☐	☐	☐	☐	☐	☐	☐	☐
**40.** Cabbage, white (boiled)	1 plate	☐	☐	☐	☐	☐	☐	☐	☐	☐
**41.** Carrot (fresh)	1 plate	☐	☐	☐	☐	☐	☐	☐	☐	☐
**42.** Carrot (boiled)	1 plate	☐	☐	☐	☐	☐	☐	☐	☐	☐
**43.** Cauliflower (raw)	1 plate	☐	☐	☐	☐	☐	☐	☐	☐	☐
**44.** Cauliflower (boiled with butter)	1 plate	☐	☐	☐	☐	☐	☐	☐	☐	☐
**45.** Chives	1 Tsp	☐	☐	☐	☐	☐	☐	☐	☐	☐
**46.** Cucumber (raw)	1 medium	☐	☐	☐	☐	☐	☐	☐	☐	☐
**47.** Garlic (raw)	1 clove	☐	☐	☐	☐	☐	☐	☐	☐	☐
**48.** Lettuce	5 leaves	☐	☐	☐	☐	☐	☐	☐	☐	☐
**49.** Mushroom (fried)	1/2 cup	☐	☐	☐	☐	☐	☐	☐	☐	☐
**50.** Onion (raw)	1 slice	☐	☐	☐	☐	☐	☐	☐	☐	☐
**51.** Parsley (leaves)	1 Tbs	☐	☐	☐	☐	☐	☐	☐	☐	☐
**52.** Horseradish	1 Tbs	☐	☐	☐	☐	☐	☐	☐	☐	☐
**53.** Pepper (cooked)	1 medium	☐	☐	☐	☐	☐	☐	☐	☐	☐
**54.** Pepper, red (raw)	1 medium	☐	☐	☐	☐	☐	☐	☐	☐	☐

Subject ID

Centre # *Community #* *Household #* *Subject #*

Subject Initials | | | |

During the past year, on average, how often have you consumed the following vegetables? (please check the appropriate box)

**Vegetables, Cont'**	**Avg Serving**	**Never, less than once/month**	**1-3/mo**	**1/wk**	**2-4/wk**	**5-6/wk**	**1/day**	**2-3/day**	**4-5/day**	**>6/day**
**55.** Potato (french fried)	1 plate	☐	☐	☐	☐	☐	☐	☐	☐	☐
**56.** Potato (boiled)	2 medium/150 g	☐	☐	☐	☐	☐	☐	☐	☐	☐
**57.** Potato (mashed)	1 plate	☐	☐	☐	☐	☐	☐	☐	☐	☐
**58.** Radish	3 pieces	☐	☐	☐	☐	☐	☐	☐	☐	☐
**59.** Tomato (raw)	1 medium	☐	☐	☐	☐	☐	☐	☐	☐	☐
**60.** Tomato (cooked)	1 cup	☐	☐	☐	☐	☐	☐	☐	☐	☐
**61.** Tomato sauce	1 cup	☐	☐	☐	☐	☐	☐	☐	☐	☐
**62.** Spinach (cooked)	1 plate	☐	☐	☐	☐	☐	☐	☐	☐	☐
**63.** Squash, summer (cooked)	1 plate	☐	☐	☐	☐	☐	☐	☐	☐	☐
**64.** String beans (boiled)	1 plate	☐	☐	☐	☐	☐	☐	☐	☐	☐
**65.** Sweet corn, (canned, drained)	1 plate	☐	☐	☐	☐	☐	☐	☐	☐	☐
**66.** Peas, green, (canned, drained)	1 plate	☐	☐	☐	☐	☐	☐	☐	☐	☐

During the past year, on average, how often have you consumed the following meat, eggs, etc.? (please check the appropriate box)

**Meat, Eggs, etc.**										
**67.** Eggs (boiled, fried)	1	☐	☐	☐	☐	☐	☐	☐	☐	☐
**68.** Beef steaks	1 piece	☐	☐	☐	☐	☐	☐	☐	☐	☐
**69.** Beef, ham (cooked)	1piece	☐	☐	☐	☐	☐	☐	☐	☐	☐
**70.** Chicken fillets (breaded, fried)	1 piece	☐	☐	☐	☐	☐	☐	☐	☐	☐
**71.** Chicken ham	1 piece	☐	☐	☐	☐	☐	☐	☐	☐	☐
**72.** Chicken with skin (cooked/fried)	1 piece	☐	☐	☐	☐	☐	☐	☐	☐	☐
**73.** Chicken without skin (cooked/fried)	1 piece	☐	☐	☐	☐	☐	☐	☐	☐	☐

Subject ID

☐☐ Centre # ☐☐☐ Community # ☐☐☐ Household # ☐☐ Subject #

Subject Initials ☐☐☐

During the past year, on average, how often have you consumed the following meat, eggs, etc.? (please check the appropriate box)

**Meat, Eggs, etc. con't**	**Avg Serving**	**Never, less than once/month**	**1-3/mo**	**1/wk**	**2-4/wk**	**5-6/wk**	**1/day**	**2-3/day**	**4-5/day**	**>6/day**
**74.** Cod fillets (breaded and fried)	1 piece	☐	☐	☐	☐	☐	☐	☐	☐	☐
**75.** Herring in cream	1 small plate	☐	☐	☐	☐	☐	☐	☐	☐	☐
**76.** Frankfurter/ Hotdog	1 medium	☐	☐	☐	☐	☐	☐	☐	☐	☐
**77.** Luncheon meat (pork)	1 slice	☐	☐	☐	☐	☐	☐	☐	☐	☐
**78.** Mackerel (smoked)	1 piece	☐	☐	☐	☐	☐	☐	☐	☐	☐
**79.** Pork, belly (no bone, boiled)	1 slice	☐	☐	☐	☐	☐	☐	☐	☐	☐
**80.** Pork ham	1 slice	☐	☐	☐	☐	☐	☐	☐	☐	☐
**81.** Pork cutlets (breaded, fried)	1 slice	☐	☐	☐	☐	☐	☐	☐	☐	☐
**82.** Sausage, Slaska (pork, cooked)	50g	☐	☐	☐	☐	☐	☐	☐	☐	☐
**83.** Sausage Krakowska (pork and beef)	50g	☐	☐	☐	☐	☐	☐	☐	☐	☐
**84.** Sausage (pork, biala)	50g	☐	☐	☐	☐	☐	☐	☐	☐	☐
**85.** Turkey, ham	1 slice	☐	☐	☐	☐	☐	☐	☐	☐	☐
**86.** Turkey (roasted)	1 slice	☐	☐	☐	☐	☐	☐	☐	☐	☐
**87.** Turkey, sausage (Szynkowa)	50g	☐	☐	☐	☐	☐	☐	☐	☐	☐
**88.** Head Cheese, white and black	50g	☐	☐	☐	☐	☐	☐	☐	☐	☐

89. Organ meat (liver,tounge,heart) — 1 piece ☐ **Never** ☐ **less than 1/month** ☐ **1/month** ☐ **2-3/month** ☐ **1/wk or more**

Subject ID

Centre #	Community #	Household #	Subject #

Subject Initials [ ] [ ] [ ]

During the past year, on average, how often have you consumed the following breads and cereals? (please check the appropriate box)

**Breads, Cereals**	**Avg Serving**	**Never, less than once/month**	**1-3/mo**	**1/wk**	**2-4/wk**	**5-6/wk**	**1/day**	**2-3/day**	**4-5/day**	**>6/day**
**90.** Wheat bread	1 slice	☐	☐	☐	☐	☐	☐	☐	☐	☐
**91.** Rice (boiled)	2 Tbs	☐	☐	☐	☐	☐	☐	☐	☐	☐
**92.** Rye, brown bread	1 slice	☐	☐	☐	☐	☐	☐	☐	☐	☐
**93.** Wheat rolls (Kajzerki)	1	☐	☐	☐	☐	☐	☐	☐	☐	☐
**94.** Wheat rolls (Wroclawskie)	1 slice	☐	☐	☐	☐	☐	☐	☐	☐	☐
**95.** Wheat-rye bread/ White bread	1 slice	☐	☐	☐	☐	☐	☐	☐	☐	☐
**96.** Wheat-rye bread with sunflower seeds	1 plate	☐	☐	☐	☐	☐	☐	☐	☐	☐
**97.** Cold Cereal (Cornflakes)	25g	☐	☐	☐	☐	☐	☐	☐	☐	☐
**98.** Pasta (cooked)	1 plate	☐	☐	☐	☐	☐	☐	☐	☐	☐

During the past year, on average, how often have you consumed the following mixed dishes? (please check the appropriate box)

**Mixed Dishes**	**Avg Serving**	**Never, less than once/month**	**1-3/mo**	**1/wk**	**2-4/wk**	**5-6/wk**	**1/day**	**2-3/day**	**4-5/day**	**>6/day**
**99.** Baked beans with meat	1 plate	☐	☐	☐	☐	☐	☐	☐	☐	☐
**100.** Beef and pork minced cutlets, fried	1 medium	☐	☐	☐	☐	☐	☐	☐	☐	☐
**101.** Broth	1 plate/400 g	☐	☐	☐	☐	☐	☐	☐	☐	☐
**102.** Buckwheat groats (boiled)	1 plate	☐	☐	☐	☐	☐	☐	☐	☐	☐
**103.** Cabbage leaves, stuffed	1 plate	☐	☐	☐	☐	☐	☐	☐	☐	☐
**104.** Chicken pate (canned)	1 slice	☐	☐	☐	☐	☐	☐	☐	☐	☐
**105.** Pearl barley groats, boiled	1 plate	☐	☐	☐	☐	☐	☐	☐	☐	☐
**106.** Soup with vegetables	1 plate/400 g	☐	☐	☐	☐	☐	☐	☐	☐	☐

Subject ID

Centre #	Community #	Household #	Subject #

Subject Initials

During the past year, on average, how often have you consumed the following mixed dishes? (please check the appropriate box)

Mixed Dishes con't	Avg Serving	Never, less than once/month	1-3/mo	1/wk	2-4/wk	5-6/wk	1/day	2-3/day	4-5/day	>6/day
**107.** Soup, Krupnik with pearl barley groats	1 plate/400 g	☐	☐	☐	☐	☐	☐	☐	☐	☐
**108.** Soup, Zurek sour rye	1 plate/400 g	☐	☐	☐	☐	☐	☐	☐	☐	☐
**109.** Soup, milk with rolled oats	1 plate/400 g	☐	☐	☐	☐	☐	☐	☐	☐	☐
**110.** Soup, tomato	1 plate/400 g	☐	☐	☐	☐	☐	☐	☐	☐	☐
**111.** Soup, sauerkraut	1 plate/400 g	☐	☐	☐	☐	☐	☐	☐	☐	☐
**112** Soup, white bean	1 plate/400 g	☐	☐	☐	☐	☐	☐	☐	☐	☐
**113.** Polish dumplings with meat	1 plate	☐	☐	☐	☐	☐	☐	☐	☐	☐
**114.** Sauerkraut with sausage and meat, (Bigos, stewed)	1 plate	☐	☐	☐	☐	☐	☐	☐	☐	☐
**115.** Shantung cabbage, salad with mayonnaise	1 small plate	☐	☐	☐	☐	☐	☐	☐	☐	☐
**116.** Sauerkraut salad	1 plate	☐	☐	☐	☐	☐	☐	☐	☐	☐
**117.** Dumplings with potato filling (Ruskie, boiled)	1 plate	☐	☐	☐	☐	☐	☐	☐	☐	☐
**118.** Lettuce with sour cream salad	1 plate	☐	☐	☐	☐	☐	☐	☐	☐	☐
**119.** Vegetable salad, cooked with mayonnaise	1 plate	☐	☐	☐	☐	☐	☐	☐	☐	☐

During the past year, on average, how often have you consumed the following beverages? (please check the appropriate box)

Beverages	Avg Serving	Never, less than once/month	1-3/mo	1/wk	2-4/wk	5-6/wk	1/day	2-3/day	4-5/day	>6/day
**120.** Orange juice	1 glass	☐	☐	☐	☐	☐	☐	☐	☐	☐
**121.** Raspberry juice	1 glass	☐	☐	☐	☐	☐	☐	☐	☐	☐

Subject ID

Centre # *Community #* *Household #* *Subject #*

Subject Initials | | | |

During the past year, on average, how often have you consumed the following beverages? (please check the appropriate box)

**Beverages, con't**	**Avg Serving**	**Never, less than once/month**	**1-3/mo**	**1/wk**	**2-4/wk**	**5-6/wk**	**1/day**	**2-3/day**	**4-5/day**	**>6/day**
**122.** Carrot juice	1 glass	☐	☐	☐	☐	☐	☐	☐	☐	☐
**123.** Apple juice	1 glass	☐	☐	☐	☐	☐	☐	☐	☐	☐
**124.** Grapefruit juice	1 glass	☐	☐	☐	☐	☐	☐	☐	☐	☐
**125.** Blackcurrent juice	1 glass	☐	☐	☐	☐	☐	☐	☐	☐	☐
**126.** Multifruit juice from Polish fruits	1 glass	☐	☐	☐	☐	☐	☐	☐	☐	☐
**127.** Multifruit juice from exotic fruits	1 glass	☐	☐	☐	☐	☐	☐	☐	☐	☐
**128.** Fruits drink	1 glass	☐	☐	☐	☐	☐	☐	☐	☐	☐
**129.** Soft drink (regular)	1 glass	☐	☐	☐	☐	☐	☐	☐	☐	☐
**130.** Soft drink (low calorie)	1 glass	☐	☐	☐	☐	☐	☐	☐	☐	☐
**131.** Coffee	1 glass	☐	☐	☐	☐	☐	☐	☐	☐	☐
**132.** Black tea	1 glass	☐	☐	☐	☐	☐	☐	☐	☐	☐
**133.** Herbal/green	1 glass	☐	☐	☐	☐	☐	☐	☐	☐	☐
**134.** Beer	1 bottle/330 mL	☐	☐	☐	☐	☐	☐	☐	☐	☐
**135.** Wine, red	1 glass	☐	☐	☐	☐	☐	☐	☐	☐	☐
**136.** Vodka	1 glass	☐	☐	☐	☐	☐	☐	☐	☐	☐

During the past year, on average, how often have you consumed the following snacks? (please check the appropriate box)

**Snacks**	**Avg Serving**	**Never, less than once/month**	**1-3/mo**	**1/wk**	**2-4/wk**	**5-6/wk**	**1/day**	**2-3/day**	**4-5/day**	**>6/day**
**137.** Milk chocolate	1 bar	☐	☐	☐	☐	☐	☐	☐	☐	☐
**138.** Bitter chocolate	1 bar	☐	☐	☐	☐	☐	☐	☐	☐	☐

Subject ID

Centre #	*Community #*	*Household #*	*Subject #*

Subject Initials

During the past year, on average, how often have you consumed the following snacks? (please check the appropriate box)

**Snacks, con't**	**Avg Serving**	**Never, less than once/month**	**1-3/mo**	**1/wk**	**2-4/wk**	**5-6/wk**	**1/day**	**2-3/day**	**4-5/day**	**>6/day**
**139.** Biscuits	small pack/50 g									
**140.** Yeast cake	1 slice									
**141.** Short-cake	1 slice									
**142.** Gingerbread cake	1 slice									
**143.** Sponge cake	1 slice									
**144.** Cheesecake (Krakowski)	1 slice									
**145.** Honey	1 Tbs									
**146.** Halva with vanilla	1 piece									
**147.** Drops	6									
**148.** Mayonnaise	1 Tbs									
**149.** Nuts	1/2 cup									
**150.** Raisins, dried	1/2 cup									
**151.** Sweets	1 Tbs									
**152.** Sugar	2 Tbs									
**153.** Seeds	1 Tbs									
**154.** Walnuts	1 Tbs									

Subject ID

Centre #	Community #	Household #	Subject #

Subject Initials

Vitamins

155. Do you regularly take vitamin pills? ☐ No ☐ Yes

156. Do you regularly take mineral pills? ☐ No ☐ Yes

If yes for how many years and how often have you taken?

	< 1 year		1-3 years		4-6 years		> 6 years	
	mostly	occasionaly	mostly	occasionaly	mostly	occasionaly	mostly	occasionaly
**157 .** Vitamin A								
**158.** Vitamin D								
**159.** Vitamin E								
**160.** Vitamin B complex								
**161.** Vitamin C								
**162.** Calcium								
**163.** Iron								
**164.** Zinc								
**165.** Multiviamin /Mineral supplement								

What type of cooking oil is usually used at home and what is the frequency of consumption?

	Avg Serving	Never, less than	1-3/mo	1/wk	2-4/wk	5-6/wk	1/day	2-3/day	4-5/day	>6/day
**166.** Soybean	1/2 cup									
**167.** Corn	1/2 cup									
**168.** Vegetable	1/2 cup									
**169.** Sunflower	1/2 cup									
**170.** Canola	1/2 cup									
**171.** Cottonseed	1/2 cup									
**172.** Olive	1/2 cup									

173. Name of Interviewer: ***(please print)*** ________ *First Initial* ____________________ *Last Name*

Interviewer Code:
